# Child undernutrition in Kenya: trend analyses from 1993 to 2008–09

**DOI:** 10.1186/1471-2431-14-5

**Published:** 2014-01-13

**Authors:** Dennis J Matanda, Maurice B Mittelmark, Dorcus Mbithe D Kigaru

**Affiliations:** 1 Department of Health Promotion and Development, University of Bergen, P.O.Box 7807, NO-5020, Christiesgt. 13 Bergen, Norway; 2 Department of Foods, Nutrition and Dietetics, Kenyatta University, Nairobi, Kenya

**Keywords:** Undernutrition, Wasting, Stunting, Underweight, Trends, Demographic and Health Survey, Kenya

## Abstract

**Background:**

Research on trends in child undernutrition in Kenya has been hindered by the challenges of changing criteria for classifying undernutrition, and an emphasis in the literature on international comparisons of countries’ situations. There has been little attention to within-country trend analyses. This paper presents child undernutrition trend analyses from 1993 to 2008–09, using the 2006 WHO criteria for undernutrition. The analyses are decomposed by child’s sex and age, and by maternal education level, household Wealth Index, and province, to reveal any departures from the overall national trends.

**Methods:**

The study uses the Kenya Demographic and Health Survey data collected from women aged 15–49 years and children aged 0–35 months in 1993, 1998, 2003 and 2008–09. Logistic regression was used to test trends.

**Results:**

The prevalence of wasting for boys and girls combined remained stable at the national level but declined significantly among girls aged 0–35 months (p < 0.05). While stunting prevalence remained stagnant generally, the trend for boys aged 0–35 months significantly decreased and that for girls aged 12–23 months significantly increased (p < 0.05). The pattern for underweight in most socio-demographic groups showed a decline.

**Conclusion:**

The national trends in childhood undernutrition in Kenya showed significant declines in underweight while trends in wasting and stunting were stagnant. Analyses disaggregated by demographic and socio-economic segments revealed some significant departures from these overall trends, some improving and some worsening. These findings support the importance of conducting trend analyses at detailed levels within countries, to inform the development of better-targeted childcare and feeding interventions.

## Background

Worldwide, about 2.2 million children die annually, with poor nutritional status as an underlying cause [1]. Global statistics for surviving undernourished children indicate that approximately 171 million children are chronically undernourished (stunted), 60 million are acutely undernourished (wasted), and 100 million are underweight [2]. Undernutrition is not only linked to child mortality but also to poor functional development of the child. Undernourished children are highly susceptible to common childhood ailments like diarrhea, respiratory infections and worm infestations. Recurrence of such ailments falters a child’s physical, behavioral, motor and cognitive development, and also compromises her/his health and functioning in adulthood [3]. Combatting child undernutrition is obviously crucial, and its complexity makes it hard to tackle. It results not only from macronutrient deficiencies (protein, fat and carbohydrate) but also from micronutrient deficiencies (trace minerals and vitamins), among which zinc deficiency is particularly deleterious to children’s normal growth [4]. Therefore, different aspects of food deprivation (quantity, quality and food group diversity) lead to different manifestations of undernutrition (wasting, stunting and underweight). Consequently, child undernutrition is a multidimensional problem that defies simple solutions. There is a fundamental need to better understand the public health dimensions of the problem, to provide a foundation for precisely targeted interventions in local contexts.

The burden of child undernutrition is unsurprisingly greatest in the world’s poorest countries, especially in sub-Saharan Africa and Asia [5]. This is a highly salient issue in Kenya, which is among the 20 countries that account for 80% of the world’s chronically undernourished children [6]. The most recent Kenyan national prevalence estimates are 35% for stunting, 7% for wasting and 16% for underweight [7,8].

A child who experiences a chronic shortage of appropriate types and quantities of food is likely to grow in height/length more slowly than expected for children of the same sex and age. Such a shortfall in growth, termed ‘stunting’, is a classical indicator of underlying child undernutrition. A child who experiences acute food shortage and/or infection is likely to gain weight more slowly than expected for children of the same sex and height/length. Such a shortfall in growth is termed ‘wasting’ , which is also a classical indicator of underlying undernutrition. Underweight is a composite indicator of stunting and wasting and thus an overall indicator of the extent of child undernutrition [9,10]. Underweight however is not a very useful indicator for interventions as it does not differentiate the extent of stunting and wasting.

According to the World Health Organisation (WHO) 2006 classification of child undernutrition, children with a Z-score below −2 Standard Deviations (SD) of the median for weight-for-height/length (WHZ) or (WLZ), height/length-for-age (HAZ) or (LAZ) and weight-for-age (WAZ) are classified as wasted, stunted and underweight respectively. Children with a Z-score below −3 SD of the median are classified as severely undernourished, while those with a Z-score between −2 SD and −3 SD are classified as being moderately undernourished. Those with a Z-score between −1 SD and −2 SD are classified as mildly undernourished [9].

There is a tendency in the literature to define and describe child undernutrition at an aggregate level, for example by reporting national prevalence for all children ages 0–59 months, without differentiation by age, sex and other factors. Yet, important differentiations do exist for specific demographic and socio-economic segments in the under-five population and nutrition interventions are correspondingly specific. In sub-Saharan Africa, boys have consistently posted higher rates of stunting compared to girls [11]. Many (sometimes contradicting) reasons have been hypothesized to explain the sex difference, such as gender-differentiated feeding practices [12,13]. It is also postulated that girls are physically less active and therefore spend less energy compared to boys, and that boys are more vulnerable to acute respiratory infection and diarrhoea [14].

Undernutrition is most critical during the first two years of life, especially stunting, after which it is difficult to restore normal growth [15]. During this early period, poor infant and young nutrition and care practices coupled with infectious diseases increase the probability of child undernutrition [16]. Studies conducted in developing countries indicate that exclusive breastfeeding is not common, with complementary foods introduced very early [17,18]. This leads to faltering child growth [19].

Level of maternal education has been documented as a determining factor in child undernutrition. In an environment with sufficient resources, mothers with education are more likely to utilize modern health care and have good health care knowledge and reproductive behaviours [20,21]. Maternal education does not, however, automatically impart nutrition knowledge, and thus mothers with education may still have undernourished children.

Given this background, it cannot be assumed that international or national trends reflect sub-group trends with validity; it is an empirical question requiring appropriate sub-group analyses. This study therefore aimed to describe time trends in child undernutrition prevalence in Kenya, with overall trends decomposed by age, province^a^, urban/rural residence, maternal education level and Wealth Index (WI), for boys and girls separately. Previous studies which have examined sub-groups in Kenya are inadequate for today’s needs for one or more of these reasons: the design was a single cross-sectional survey and therefore not useful to define trends over time; the study sample was not nationally representative data; the study was conducted before 2006 and hence used out-dated reference standards for child growth [17,22-26].

The present study addresses these limitations, by undertaking trend analyses of stunting, wasting and underweight, in defined sub-groups in Kenya, and using the 2006 WHO child growth standards in the analysis of data collected from four cross-sectional surveys conducted in 1993, 1998, 2003 and 2008–09. The surveys used identical methods, making their results comparable.

## Methods

### Data

This study used data from the Kenya Demographic and Health Survey (KDHS), a series of national cross-sectional surveys conducted in 1993, 1998, 2003 and 2008–09 (data from KDHS earlier than 1993 are not used). These datasets are publicly accessible through application to MEASURE DHS^b^[26]. In all survey years, data were collected using identical questionnaire items for women of reproductive age 15–49 years old. In all four surveys, a standard child anthropometry protocol was used. Children 0–59 months were weighed using scales fitted with a digital screen and measured for height using a measuring board. Weight was recorded in kilograms and height/length in centimeters. Children younger than 24 months were measured lying down on the board (recumbent length), while standing height was recorded for older children. Extensive information on data collection and management has been published elsewhere [7,27-30].

Table 1 shows the two-stage sampling design used by the Kenya Demographic and Health Survey. The first stage involved selecting data collection points (clusters) from the national master sample frame and then households were systematically sampled from the selected clusters with women of ages 15–49 years eligible for interview [7,27-29].

**Table 1:** Sampling design, KDHS

	**1993**	**1998**	**2003**	**2008-09**
Clusters Selected	536	536	400	400
Households Selected	8805	9465	9865	9936
Women Interviewed	7540	7881	8195	8444
Response Rate	95%	96%	94%	96%

To enable a trend analysis, variables of interest were identified in the base year data file (1993). Thereafter, data files were sorted by their identification variables and the four cross-sectional datasets of 1993, 1998, 2003 and 2008–09 were merged into a single data file. Besides examining trends for the samples as wholes, sub-group analyses were undertaken, separately for boys and girls, by age, province, residence, maternal education and WI. In each trend analysis, logistic regression was used to test the null hypothesis that the regression coefficient *β* for survey year was not significantly different from zero, using the equation:

$$\log\left(p/1-p\right)=\beta_{0}+\beta_{\mathrm{survey}\;\mathrm{year}}\cdot \;\mathrm{survey}\;\mathrm{year}$$

Due to lack of anthropometry data for children older than 36 months in the 1998 survey, the analysis reported in this paper was restricted to children aged 0–35 months. This allowed comparability of trends across the four surveys from 1993 to 2009. The age categories analyzed were 0–5 months, 6–11 months, 12–23 months and 24–35 months. During the 1993 and 1998 survey years, KDHS did not collect data in North-Eastern province. Consequently, North-Eastern province was excluded in the analysis in order to allow comparison of prevalence across all the four survey years. Provinces included in the analysis include Nairobi, Central, Coast, Eastern, Nyanza, Rift-Valley and Western.

Self-reported maternal education level was categorized as no education, incomplete primary, complete primary and incomplete secondary education. Sample size limitations in the 1993 survey for the higher education category were overcome by combining the complete secondary education and higher education categories in the analyses presented in this paper.

Standard of living measurement involved classification of children into quintiles based on the household Wealth Index. This is a proxy for standard of living based on household ownership of assets and housing quality. Each asset is assigned a factor score generated through principal component analysis, with the scores summed and standardized. All individuals are assigned the score and the quintile (poorest, poorer, middle, richer and richest) of their household [31].

### Child anthropometry

In assessing children’s nutritional status, wasting (low weight-for-length/height), stunting (low length/height-for-age) and underweight (low weight-for-age) were used as the three indicators of child undernutrition. In conformity with the recommended World Health Organization (WHO) child growth standards of 2006, the SPSS syntax file ‘igrowup_DHSind.sps’ was used to calculate Z-scores for the three anthropometric indicators. Children were considered wasted, stunted or underweight if their WHZ/WLZ, HAZ/LAZ and WAZ score was less than −2 SD respectively. Extreme Z-scores considered to be biologically implausible were flagged and not used in the analysis if WHZ/WLZ score was less than −5 SD or greater than 5 SD, HAZ/LAZ score was less than −6 SD or greater than 6 SD and WAZ score was less than −6 SD or greater than 5 SD [32,33].

### Analysis

SPSS for windows version 19 was used to conduct the analyses. The design effect parameters ‘sampling weight’, ‘sample domain’ and ‘sample cluster’ [32] were incorporated using SPSS’ Complex Samples Module. In line with recommendations that emphasize provision of levels of uncertainty in the estimates of undernutrition [33], 95% confidence intervals (C.I.) for the prevalence estimates were computed and are presented in Tables 2, 3, 4. Logistic regression was used to test trends. This involved modeling change in undernutrition prevalence regressed on time (the four survey years) with probability values for Wald F tests less than 0.05 considered significant (Tables 2, 3, 4). It is important to note that in the Tables, the 95% C.I. are calculated separately for each prevalence estimate and are not associated with the Wald F statistics that were generated by the logistic regression tests for trends.

**Table 2:** Wasting trends by age, province, residence, maternal education and wealth index, KDHS

		**1993**			**1998**			**2003**			**2008-09**					
	**Sex**	**n**	**%**	**C.I.**	**n**	**%**	**C.I.**	**n**	**%**	**C.I.**	**n**	**%**	**C.I.**	**Wald F**	**P-value**	**Trend**
**Total**	M/F	2,969	8.4	7.2-9.8	2,921	8.7	7.6-10.0	3,020	7.2	6.0-8.5	3,028	7.4	6.1-8.9	2.206	0.138	
	M	1,789	9.1	7.5-10.9	1,501	9.2	7.7-10.9	1,818	8.6	7.0-10.5	1,807	8.6	7.0-10.5	0.259	0.611	
	F	1,180	7.3	5.8-9.3	1,420	8.2	6.7-10.1	1,202	5.0	3.8-6.5	1,221	5.6	4.3-7.4	5.338	0.021	
**Age**																
0-5 months	M	275	9.7	6.3-14.7	273	9.9	6.5-14.6	342	10.0	6.9-14.4	308	8.0	5.2-12.2	0.293	0.589	
	F	160	8.3	4.8-14.1	179	8.6	5.0-14.4	170	5.6	2.9-10.6	142	12.8	7.8-20.2	0.391	0.532	
6-11 months	M	367	10.4	7.3-14.6	293	14.9	10.4-20.9	366	10.0	7.1-13.9	373	13.2	8.1-20.8	0.104	0.747	
	F	179	7.0	4.0-12.0	232	9.9	6.4-15.2	221	4.7	2.6-8.3	185	6.5	3.2-13.0	0.056	0.812	
12-23 months	M	648	10.2	7.4-14.0	528	9.1	6.8-12.1	638	8.9	6.6-12.0	583	5.3	3.6-7.8	5.714	0.017	
	F	360	8.9	5.9-13.3	489	9.8	7.1-13.3	385	6.4	4.3-9.5	430	4.2	2.7-6.6	8.977	0.003	
24-35 months	M	500	6.2	4.3-9.0	407	4.6	2.8-7.4	472	6.1	4.0-9.4	543	9.2	5.8-14.3	3.139	0.077	
	F	482	6.0	4.0-8.8	520	5.9	4.0-8.6	426	3.5	2.0-6.1	464	4.4	2.4-8.0	1.431	0.232	
**Province**																
Nairobi	M	58	4.8	1.3-16.3	120	13.8	8.3-22.2	111	4.6	2.1-10.1	84	9.7	4.0-21.7	0.010	0.922	
	F	43	0.0		68	10.8	3.8-26.9	88	4.9	1.2-18.2	73	2.9	0.6-12.5	0.089	0.766	
Central	M	199	3.5	1.4-8.3	127	9.2	5.2-15.9	182	5.2	2.8-9.3	122	5.2	2.5-10.5	0.213	0.645	
	F	159	4.6	2.0-10.2	142	8.2	3.7-17.1	145	6.6	3.6-11.6	110	7.2	3.6-14.1	0.409	0.524	
Coast	M	146	13.7	9.5-19.4	126	7.4	4.4-12.2	167	8.2	5.0-13.2	173	13.7	8.9-20.5	0.012	0.913	
	F	93	13.9	8.3-22.3	112	6.4	3.7-10.8	97	4.7	2.1-10.0	108	11.8	7.2-18.7	0.172	0.679	
Eastern	M	344	10.5	7.0-15.6	230	6.8	4.0-11.3	321	7.9	4.7-12.8	268	4.8	2.7-8.2	3.981	0.048	
	F	248	11.7	7.3-18.0	259	6.4	3.3-12.1	184	2.7	1.0-7.5	233	5.7	2.8-11.1	3.583	0.060	
Nyanza	M	292	9.6	6.7-13.6	312	12.9	9.0-18.2	278	6.3	3.7-10.6	385	6.1	4.1-9.0	6.403	0.012	
	F	189	5.2	2.6-10.0	297	11.2	8.1-15.4	201	1.5	0.5-5.0	222	4.5	2.3-8.7	3.496	0.063	
Rift-Valley	M	418	10.9	7.2-16.3	396	7.8	5.4-11.2	520	12.6	9.0-17.5	533	13.4	10.0-17.8	1.794	0.182	
	F	268	7.5	4.8-11.6	340	7.7	5.3-11.2	323	6.3	3.9-10.2	318	6.4	3.6-11.2	0.375	0.541	
Western	M	333	6.8	4.4-10.4	193	6.9	4.0-11.8	239	8.3	4.5-14.9	242	3.8	1.6-8.8	0.777	0.379	
	F	181	4.2	2.0-8.6	203	7.2	4.0-12.6	163	7.8	4.7-12.6	158	1.5	0.5-4.8	1.199	0.275	
**Residence**																
Urban	M	192	6.4	3.8-10.7	283	7.9	4.8-12.7	324	5.6	3.6-8.6	305	7.4	4.7-11.5	0.001	0.973	
	F	135	5.9	3.0-11.5	244	7.9	4.8-12.9	222	3.8	1.7-8.6	244	3.5	1.8-6.6	3.611	0.058	
Rural	M	1,598	9.4	7.7-11.4	1,218	9.5	7.8-11.4	1,494	9.3	7.4-11.5	1,502	8.8	7.0-11.1	0.182	0.670	
	F	1,046	7.5	5.8-9.6	1,177	8.3	6.6-10.3	980	5.2	3.9-7.0	977	6.2	4.6-8.3	2.982	0.085	
**Maternal Education**																
No education	M	309	16.2	11.8-21.9	164	7.6	4.2-13.5	236	18.4	13.0-25.5	182	18.6	13.4-25.3	1.126	0.289	
	F	206	12.4	8.4-17.8	144	11.3	6.8-18.4	155	6.0	2.7-12.6	114	8.7	4.7-15.4	2.498	0.115	
Incomplete primary	M	710	9.4	7.1-12.2	557	12.2	9.5-15.4	678	11.1	8.5-14.4	666	8.5	6.1-11.6	0.296	0.587	
	F	476	6.6	4.5-9.7	548	8.6	6.3-11.7	456	5.7	3.8-8.4	411	4.8	2.6-8.4	2.006	0.157	
Complete primary	M	366	8.0	5.6-11.4	377	9.1	6.3-13.1	519	3.4	2.0-5.5	562	6.6	4.5-9.7	1.994	0.158	
	F	226	6.4	3.6-11.0	370	8.6	5.7-12.8	326	5.1	3.1-8.3	363	8.9	5.6-13.8	0.186	0.666	
Incomplete secondary	M	363	4.4	2.4-7.8	148	7.3	3.7-14.2	165	5.3	2.5-11.0	157	7.4	3.7-14.1	1.048	0.306	
	F	227	4.6	2.5-8.0	132	5.1	2.2-11.6	97	4.7	1.8-11.6	111	4.1	1.5-10.7	0.024	0.878	
Secondary +	M	42	1.1	0.2-7.9	256	4.8	2.4-9.1	220	5.3	2.8-9.7	240	6.7	3.9-11.5	1.718	0.190	
	F	45	10.5	3.7-26.5	225	6.6	3.8-11.3	168	1.9	0.7-4.7	223	1.2	0.4-3.4	14.170	0.000	
**Wealth Index**																
Poorest	M	403	15.2	11.3-20.2	378	11.3	8.1-15.6	442	10.7	7.2-15.7	433	13.0	9.7-17.2	0.601	0.439	
	F	279	7.6	4.5-12.4	345	9.3	6.5-13.1	263	6.9	4.2-11.2	264	7.6	4.5-12.6	0.086	0.769	
Poorer	M	395	10.1	7.3-13.7	302	12.2	8.8-16.5	391	10.8	7.4-15.4	415	9.6	5.1-17.4	0.053	0.818	
	F	240	10.9	7.4-15.7	303	8.0	5.2-12.2	269	5.2	3.0-9.1	243	7.0	3.4-13.7	1.858	0.173	
Middle	M	385	6.6	4.4-9.6	288	5.9	3.2-10.4	347	7.5	4.8-11.3	350	5.8	3.1-10.6	0.010	0.922	
	F	227	8.0	4.7-13.3	258	9.8	6.3-14.8	236	3.5	1.8-6.6	240	4.9	2.7-8.9	3.945	0.047	
Richer	M	343	7.8	4.9-12.2	271	6.0	3.6-9.9	316	7.4	4.5-11.7	305	7.1	4.5-10.9	0.028	0.868	
	F	231	5.1	2.6-9.8	258	8.1	4.7-13.5	213	4.8	2.4-9.1	246	5.7	3.1-10.3	0.036	0.849	
Richest	M	264	3.4	1.8-6.4	262	9.5	6.1-14.5	323	5.6	3.5-8.8	305	5.6	3.1-9.8	0.100	0.752	
	F	204	4.7	2.5-8.8	256	5.6	3.2-9.9	221	4.1	1.8-9.1	229	2.5	1.1-5.5	1.983	0.160	

C.I, 95% confidence intervals; Secondary +, complete secondary and/or higher education; , significant decreasing trend.

**Table 3:** Stunting trends by age, province, residence, maternal education and wealth index, KDHS

		**1993**			**1998**			**2003**			**2008-09**					
	**Sex**	**n**	**%**	**C.I.**	**n**	**%**	**C.I.**	**n**	**%**	**C.I.**	**n**	**%**	**C.I.**	**Wald F**	**P-value**	**Trend**
**Total**	M/F	2,996	39.5	37.3-41.7	2,951	37.1	34.9-39.2	3,033	36.1	33.9-38.4	3,051	36.5	33.6-39.5	2.681	0.102	
	M	1,805	41.7	38.9-44.7	1,511	39.5	36.8-42.2	1,827	38.8	36.0-41.6	1,822	36.9	33.7-40.2	4.634	0.032	
	F	1,191	36.0	32.8-39.3	1,440	34.5	31.7-37.5	1,206	32.1	29.0-35.4	1,229	35.9	31.7-40.3	0.089	0.766	
**Age**																
0-5 months	M	276	20.5	15.4-26.7	286	17.9	13.4-23.6	355	17.8	13.4-23.2	320	14.3	9.0-22.1	2.288	0.131	
	F	167	10.6	6.4-16.9	190	15.7	10.7-22.5	169	14.5	9.5-21.6	149	15.5	8.9-25.8	0.135	0.713	
6-11 months	M	372	24.9	20.5-29.9	290	24.5	19.1-30.7	363	21.5	16.9-26.9	372	27.3	21.8-33.6	0.011	0.917	
	F	178	22.9	16.4-31.0	239	18.6	13.7-24.7	223	16.0	11.1-22.4	184	24.3	16.6-34.0	0.108	0.742	
12-23 months	M	655	52.9	48.0-57.8	528	50.5	45.9-55.1	642	50.4	45.5-55.3	580	49.7	43.8-55.5	0.488	0.485	
	F	360	31.3	26.1-37.0	489	36.9	32.5-41.7	386	38.7	33.3-44.3	432	40.1	31.5-49.4	4.179	0.041	
24-35 months	M	502	51.3	46.1-56.5	407	51.0	45.5-56.5	467	52.2	46.8-57.6	550	43.1	37.1-49.4	3.242	0.072	
	F	485	53.1	47.6-58.5	522	46.4	41.6-51.3	428	41.5	35.9-47.4	464	43.1	37.6-48.7	9.880	0.002	
**Province**																
Nairobi	M	65	61.7	46.3-75.1	120	32.3	24.0-41.8	111	29.2	22.0-37.6	85	30.4	20.9-41.9	8.505	0.005	
	F	46	18.2	10.7-29.1	66	27.8	15.4-44.9	89	18.4	11.8-27.6	73	30.6	20.8-42.6	0.880	0.351	
Central	M	202	45.2	36.0-54.7	127	41.9	33.5-50.8	188	41.2	33.7-49.2	126	37.2	29.0-46.1	1.317	0.253	
	F	160	34.4	27.1-42.4	145	32.4	21.8-45.2	145	31.0	23.9-39.0	110	24.1	15.3-35.8	2.149	0.145	
Coast	M	145	42.0	34.4-50.0	128	48.5	36.7-60.5	168	41.0	32.6-50.0	174	39.9	34.3-45.8	0.505	0.478	
	F	96	47.3	37.8-57.0	113	41.0	32.4-50.2	98	41.3	31.4-51.9	108	42.2	28.1-57.7	0.224	0.636	
Eastern	M	345	46.3	39.5-53.2	238	42.3	36.5-48.4	319	42.6	35.0-50.5	269	43.1	37.1-49.4	0.457	0.500	
	F	246	45.5	37.5-53.8	261	38.9	32.5-45.7	189	33.7	24.4-44.5	235	39.7	31.9-48.1	1.249	0.265	
Nyanza	M	297	40.6	34.7-46.8	310	33.8	28.0-40.1	279	33.2	27.3-39.7	381	30.8	26.1-35.9	5.347	0.022	
	F	191	37.9	31.2-45.1	307	33.5	27.4-40.1	204	32.2	25.1-40.2	220	35.0	28.1-42.6	0.262	0.609	
Rift-Valley	M	420	39.1	32.9-45.6	393	39.8	36.1-43.7	516	40.3	35.0-45.8	545	41.2	33.5-49.4	0.178	0.673	
	F	269	27.6	22.0-34.0	346	32.9	27.4-38.9	318	32.7	27.0-38.9	326	38.3	27.3-50.6	2.240	0.136	
Western	M	331	35.3	29.6-41.5	196	41.3	33.1-50.0	245	38.0	31.2-45.3	242	30.1	25.5-35.2	1.235	0.268	
	F	184	33.6	25.9-42.2	201	33.4	26.9-40.6	165	32.0	24.1-41.1	158	32.9	25.1-41.8	0.038	0.846	
**Residence**																
Urban	M	199	41.9	32.7-51.6	281	30.4	24.9-36.5	327	35.3	29.4-41.7	307	29.5	23.7-36.0	2.647	0.105	
	F	138	17.7	12.0-25.5	247	27.7	20.9-35.6	223	24.4	19.5-30.0	244	25.7	16.4-37.7	0.452	0.502	
Rural	M	1,606	41.7	38.7-44.8	1,230	41.5	38.6-44.5	1,500	39.5	36.4-42.7	1,515	38.4	34.9-42.1	2.300	0.130	
	F	1,052	38.4	35.0-41.9	1,193	36.0	32.9-39.2	983	33.9	30.3-37.6	985	38.4	34.1-43.0	0.057	0.812	
**Maternal Education**																
No education	M	313	44.0	37.5-50.8	166	49.3	39.4-59.2	233	41.7	32.8-51.2	190	36.8	29.1-45.3	1.975	0.161	
	F	209	42.9	35.3-50.9	144	43.8	34.3-53.8	152	41.3	31.3-52.0	114	44.0	29.9-59.2	0.000	0.998	
Incomplete primary	M	716	48.8	44.6-53.0	573	44.7	40.4-49.1	683	42.1	37.9-46.4	664	41.5	36.2-47.1	5.069	0.025	
	F	475	41.1	36.0-46.5	561	39.1	34.9-43.5	457	35.9	30.6-41.5	410	40.6	34.6-47.0	0.180	0.671	
Complete primary	M	367	40.4	34.9-46.2	380	37.8	32.7-43.2	526	38.6	33.9-43.5	565	40.1	33.9-46.8	0.004	0.951	
	F	228	31.8	25.5-38.8	371	38.0	32.4-43.8	330	35.8	30.5-41.5	369	34.7	28.7-41.2	0.041	0.839	
Incomplete secondary	M	367	29.3	23.5-35.9	139	31.9	23.8-41.2	165	39.4	30.2-49.4	156	27.8	20.0-37.3	0.229	0.633	
	F	232	24.3	18.7-31.0	135	27.5	20.5-35.8	96	21.1	13.0-32.3	112	27.4	18.3-38.7	0.043	0.836	
Secondary +	M	42	24.0	12.6-40.9	254	28.0	22.7-34.0	219	25.2	19.7-31.5	247	23.1	16.7-31.1	0.700	0.403	
	F	46	31.2	18.1-48.2	228	16.0	11.6-21.7	171	12.8	8.8-18.2	224	29.3	20.9-39.5	1.749	0.187	
**Wealth Index**																
Poorest	M	405	48.6	42.8-54.5	384	50.6	44.5-56.6	448	45.8	40.9-50.8	450	42.9	36.8-49.3	2.568	0.110	
	F	281	44.0	37.8-50.5	353	44.0	38.1-50.1	261	40.4	33.4-47.9	268	46.6	39.8-53.4	0.066	0.797	
Poorer	M	399	43.9	38.3-49.7	303	41.7	36.0-47.5	389	38.5	33.0-44.3	411	44.7	37.6-51.9	0.003	0.960	
	F	243	37.7	31.6-44.2	307	40.9	34.8-47.3	272	35.1	29.2-41.6	247	37.4	28.8-46.9	0.174	0.677	
Middle	M	385	40.9	34.9-47.2	288	35.8	29.5-42.6	350	34.5	28.5-40.9	345	32.7	26.9-39.1	3.596	0.058	
	F	227	34.4	28.0-41.5	261	35.6	30.1-41.5	236	34.0	27.5-41.1	241	39.6	31.2-48.7	0.633	0.426	
Richer	M	346	41.7	35.9-47.9	279	38.2	32.6-44.1	312	40.1	33.8-46.7	304	28.9	22.5-36.3	5.982	0.015	
	F	232	41.8	34.8-49.1	262	31.1	24.7-38.4	211	29.2	22.9-36.5	241	32.5	23.4-43.1	1.999	0.158	
Richest	M	270	29.4	23.4-36.2	258	26.0	20.2-32.7	328	32.9	26.5-39.9	312	30.7	23.5-38.9	0.439	0.508	
	F	207	18.4	13.3-25.0	259	16.5	11.7-22.7	227	19.5	15.1-24.8	231	21.6	14.6-30.7	0.713	0.399	

C.I, 95% confidence intervals; Secondary +, complete secondary and/or higher education; , significant decreasing trend; , significant increasing trend.

**Table 4:** Underweight trends by age, province, residence, maternal education and wealth index, KDHS

		**1993**			**1998**			**2003**			**2008-09**					
	**Sex**	**n**	**%**	**C.I.**	**n**	**%**	**C.I.**	**n**	**%**	**C.I.**	**n**	**%**	**C.I.**	**Wald F**	**P-value**	**Trend**
**Total**	M/F	3,115	19.7	17.9-21.6	3,051	17.6	16.0-19.4	3,148	16.0	14.4-17.8	3,147	15.0	13.0-17.2	11.804	0.001	
	M	1,881	21.4	19.1-23.8	1,580	19.4	17.2-21.9	1,880	18.7	16.4-21.2	1,890	16.4	14.1-19.1	7.964	0.005	
	F	1,234	17.2	14.6-20.2	1,471	15.6	13.6-18.0	1,269	12.1	10.0-14.5	1,257	12.8	10.3-15.7	7.237	0.007	
**Age**																
0-5 months	M	307	13.4	9.6-18.4	309	9.3	6.1-14.0	381	9.8	6.7-13.8	352	5.1	3.0-8.4	9.369	0.002	
	F	178	7.6	4.5-12.5	195	8.9	5.4-14.4	2,001	4.4	2.2-8.6	160	5.8	2.6-12.5	1.781	0.182	
6-11 months	M	379	18.0	14.0-22.8	305	16.4	12.3-21.4	370	17.0	13.3-21.6	386	18.2	12.3-26.0	0.016	0.898	
	F	189	14.1	9.4-20.6	242	10.6	6.9-16.2	229	8.4	5.3-13.2	187	6.2	3.2-11.9	4.093	0.043	
12-23 months	M	674	26.4	22.4-30.9	546	22.1	18.4-26.1	649	22.1	18.0-26.9	597	17.9	14.5-22.0	8.317	0.004	
	F	371	17.6	13.5-22.5	504	16.8	13.4-20.8	396	15.0	11.4-19.5	439	11.4	7.5-16.8	1.720	0.190	
24-35 months	M	520	22.1	18.4-26.2	420	25.8	21.2-30.9	480	22.2	18.0-27.2	555	20.9	16.1-26.7	0.146	0.702	
	F	496	21.6	17.3-26.6	530	19.3	16.0-23.2	443	14.9	11.3-19.5	470	19.0	15.1-23.8	3.309	0.069	
**Province**																
Nairobi	M	72	11.5	4.9-24.9	121	13.6	8.2-21.8	114	6.2	3.1-12.2	93	9.2	5.2-15.7	1.198	0.277	
	F	51	2.7	0.4-15.4	72	2.6	0.4-14.7	91	4.5	2.1-9.5	80	7.3	2.7-18.4	1.330	0.252	
Central	M	201	16.0	12.1-21.0	138	8.4	4.6-14.7	188	12.3	8.4-17.8	131	12.7	5.7-25.7	0.313	0.576	
	F	165	10.8	6.5-17.2	149	10.8	5.8-19.1	154	9.0	5.3-15.0	115	10.3	5.6-18.1	0.077	0.782	
Coast	M	173	29.5	23.0-36.9	134	23.4	15.1-34.5	178	19.9	14.7-26.3	178	23.2	17.3-30.4	1.807	0.181	
	F	110	25.7	17.5-36.2	115	19.4	14.4-25.4	102	13.8	8.2-22.3	109	20.9	14.4-29.5	1.015	0.315	
Eastern	M	357	23.7	18.2-30.4	256	22.2	16.9-28.7	326	20.7	15.8-26.8	275	16.9	12.0-23.2	2.548	0.112	
	F	262	24.3	16.9-33.7	270	18.4	14.0-23.8	195	11.4	6.6-19.1	235	14.3	8.6-23.1	3.711	0.056	
Nyanza	M	308	21.6	17.4-26.6	315	22.7	17.6-28.8	291	14.6	10.1-20.8	398	14.0	9.9-19.4	6.951	0.009	
	F	193	20.9	15.1-28.1	302	20.2	14.8-27.0	214	9.1	5.2-15.5	226	10.8	7.0-16.3	10.394	0.002	
Rift-Valley	M	432	24.0	18.9-30.1	417	19.0	15.3-23.5	537	23.0	18.0-28.9	570	20.2	15.7-25.5	0.403	0.526	
	F	271	12.8	9.2-17.6	357	13.8	10.1-18.4	347	15.1	10.7-21.0	331	13.6	8.2-21.6	0.075	0.784	
Western	M	337	16.4	12.1-22.0	197	20.0	13.2-29.1	245	21.0	14.8-28.9	245	11.1	6.4-18.5	0.768	0.382	
	F	181	14.4	9.8-20.6	207	14.6	9.7-21.5	165	16.4	11.3-23.2	161	10.4	6.7-15.8	0.751	0.388	
**Residence**																
Urban	M	215	12.2	8.2-17.8	294	12.0	8.0-17.6	335	13.7	9.2-19.9	319	13.0	8.9-18.7	0.144	0.704	
	F	145	8.3	4.1-15.9	256	9.5	6.5-13.5	241	7.4	4.4-12.1	254	8.5	4.6-15.3	0.025	0.874	
Rural	M	1,665	22.6	20.1-25.2	1,286	21.2	18.7-23.9	1,544	19.7	17.2-22.6	1,570	17.1	14.5-20.1	8.309	0.004	
	F	1,089	18.4	15.5-21.6	1,215	16.9	14.5-19.6	1,028	13.2	10.8-16.0	1,003	13.8	11.1-17.1	6.301	0.012	
**Maternal Education**																
No education	M	331	30.7	25.0-37.2	174	31.1	22.3-41.6	244	32.1	25.1-40.0	197	27.4	21.1-34.8	0.233	0.629	
	F	220	27.0	20.3-35.0	148	27.0	19.5-36.0	168	19.2	13.5-26.7	115	22.2	13.9-33.4	1.540	0.215	
Incomplete primary	M	743	26.1	22.7-29.8	596	25.8	21.9-30.2	704	21.5	18.0-25.4	681	19.4	15.5-23.9	7.439	0.006	
	F	494	18.6	15.1-22.8	569	17.9	14.2-22.4	476	13.4	10.1-17.5	419	15.3	11.5-20.2	2.466	0.117	
Complete primary	M	383	16.8	13.1-21.3	395	13.8	10.7-17.7	538	15.1	11.8-19.2	590	15.5	11.4-20.6	0.034	0.854	
	F	231	11.8	7.9-17.4	384	16.1	12.6-20.4	348	12.8	8.8-18.3	376	14.2	9.8-20.1	0.023	0.880	
Incomplete secondary	M	383	10.3	7.3-14.3	152	11.0	6.5-17.9	167	13.0	7.6-21.4	164	11.0	6.4-18.3	0.239	0.625	
	F	243	11.4	7.5-16.9	139	9.4	5.3-15.9	102	4.1	1.3-12.4	115	7.1	3.7-13.3	2.837	0.093	
Secondary +	M	42	8.6	2.4-26.4	263	10.5	6.4-16.7	227	8.1	4.9-13.1	258	6.0	3.6-9.8	2.163	0.142	
	F	46	12.8	5.2-28.2	232	5.6	3.3-9.5	175	5.0	2.5-9.6	232	3.9	1.8-8.1	2.529	0.112	
**Wealth Index**																
Poorest	M	419	31.7	26.3-37.5	398	30.9	25.6-36.7	457	24.1	19.3-29.6	463	24.2	19.3-29.8	5.402	0.020	
	F	290	22.6	17.4-28.7	353	22.0	17.6-27.1	277	18.4	13.2-25.1	271	16.9	12.4-22.6	2.718	0.100	
Poorer	M	415	25.4	20.9-30.6	317	21.6	17.5-26.3	398	20.7	15.8-26.6	424	19.7	14.6-26.0	2.204	0.138	
	F	248	20.3	15.2-26.5	317	17.6	12.8-23.7	282	12.4	8.5-17.9	251	15.9	10.5-23.3	1.788	0.182	
Middle	M	396	17.5	13.9-21.7	308	14.3	10.3-19.5	359	16.9	12.8-22.0	358	10.9	7.3-15.9	3.208	0.074	
	F	237	17.6	12.7-23.9	271	16.0	11.8-21.5	243	14.5	10.4-19.8	246	17.9	11.9-26.0	0.001	0.980	
Richer	M	367	19.3	15.2-24.2	289	17.4	12.8-23.2	329	18.1	13.9-23.2	318	13.4	8.2-21.0	1.808	0.179	
	F	241	15.2	10.5-21.5	263	11.9	7.9-17.6	223	7.8	4.3-13.6	248	7.6	3.8-14.7	4.264	0.039	
Richest	M	285	8.6	5.6-12.9	268	8.1	5.2-12.3	337	11.3	7.2-17.3	326	10.4	6.0-17.4	0.691	0.406	
	F	219	8.4	5.0-13.8	269	8.2	5.4-12.3	244	6.1	3.4-10.8	241	4.9	2.6-9.2	2.238	0.135	

C.I, 95% confidence intervals; Secondary +, complete secondary and higher education; , significant decreasing trend.

## Results

### Description of the study samples

Table 5 shows the sample distributions for each year by child’s growth, sex and age, and by province, urban/rural residence, maternal education and Wealth Index. Sample sizes in the various socio-demographic groups varied considerably, affecting the comparability of the Wald F Statistics generated by logistic regression in the tests of trends (shown in Tables 2, 3, 4). This variability should be kept in mind in the examination of the data in Tables 2, 3, 4.

**Table 5:** Growth and socio-demographic characteristics of the samples, KDHS

	**1993**		**1998**		**2003**		**2008-09**	
	**n**	**%**	**n**	**%**	**n**	**%**	**n**	**%**
**Growth**								
Wasted	249	8.4	255	8.7	216	7.2	224	7.4
Stunted	1,182	39.5	1,094	37.1	1,095	36.1	1,114	36.5
Underweight	615	19.7	537	17.6	504	16.0	471	15.0
**Sex**								
Male	2,020	60.1	1,647	51.4	1,930	59.4	1,917	60.0
Female	1,343	39.9	1,559	48.6	1,320	40.6	1,281	40.0
**Age**								
0-5 months	521	15.5	523	16.3	603	18.6	516	16.1
6-11 months	593	17.6	564	17.6	615	18.9	585	18.3
12-23 months	1,124	33.4	1,097	34.2	1,080	33.2	1,048	32.8
24-35 months	1,126	33.5	1,021	31.9	952	29.3	1,049	32.8
**Province**								
Nairobi	157	4.7	213	6.7	216	6.7	181	5.6
Central	390	11.6	296	9.2	357	11.0	249	7.8
Coast	310	9.2	263	8.2	290	8.9	289	9.0
Eastern	668	19.9	546	17.0	537	16.5	514	16.1
Nyanza	526	15.6	641	20.0	510	15.7	630	19.7
Rift-Valley	769	22.9	824	25.7	924	28.4	927	29.0
Western	543	16.2	423	13.2	417	12.8	408	12.8
**Residence**								
Urban	430	12.8	600	18.7	607	18.7	591	18.5
Rural	2,934	87.2	2,606	81.3	2,644	81.3	2,608	81.5
**Maternal Education**								
No education	614	18.3	339	10.6	432	13.3	318	9.9
Incomplete primary	1,320	39.2	1,221	38.1	1,209	37.2	1,115	34.9
Complete primary	665	19.8	811	25.3	914	28.1	982	30.7
Incomplete secondary	670	19.9	306	9.6	281	8.6	283	8.8
Secondary +	94	2.8	528	16.5	416	12.8	501	15.7
**Wealth Index**								
Poorest	762	22.7	782	24.4	752	23.1	745	23.3
Poorer	702	20.9	663	20.7	695	21.4	684	21.4
Middle	672	20.0	606	18.9	620	19.1	610	19.1
Richer	657	19.5	581	18.1	571	17.6	576	18.0
Richest	570	16.9	573	17.9	614	18.9	584	18.3

Secondary +, complete secondary and/or higher education.

### Trends in wasting

National trends for boys and girls combined and for boys aged 0–35 months showed no decline in wasting across the study period (Table 2), while wasting did decrease significantly for girls from 7.3% in 1993 to 5.6% in 2008–09 (F(1, 1136) = 5.34, p < 0.021). The decline in girls was concentrated in the age group 12–23 months (F(1, 1046) = 8.98, p < 0.003), and the decline in boys was concentrated in the same age group (F(1, 1046) = 5.71, p < 0.017).

By province, a departure from the overall trends was observed in Eastern and Nyanza provinces. In Eastern province, wasting among boys decreased significantly from 10.5% in 1993 to 4.8% in 2008–9 (F(1, 172) = 3.98, p < 0.048). Boys in Nyanza province posted a significant decline in wasting from 9.6% in 1993 to 6.1% in 2008–9 (F(1, 161) = 6.40, p < 0.012). Analyses by maternal education showed that the prevalence of wasting among girls with mothers having complete secondary and/or higher education declined significantly from 10.5% to 1.2% from 1993 to 2008–9 (F(1, 611) = 14.17, p < 0.000). Trends by urban/rural residence were not statistically significant while those by WI showed girls in the middle quintile decrease from 8.0% in 1993 to 4.9% in 2008–09 (F(1, 735) = 3.95, p < 0.047).

Comparing wasting prevalence between two survey years (1993 versus 2008–09), boys recorded poor growth patterns as compared to girls. Prevalence for boys increased among 6–11 months olds (10.4% to 13.2%), boys in Rift-Valley increased (10.9% to 13.4%), and boys born to mothers with no education (16.2% to 18.6%).

### Trends in stunting

Nationally, prevalence in stunting for boys and girls combined remained stagnant across the survey years. The gender-specific trends showed boys’ trend declining from 41.7% in 1993 to 36.9% in 2008–9 (F(1, 1137) = 4.63, p < 0.032) while the trend for girls was stable (Table 3). There was a worsening trend in stunting for girls aged 12–23 months, with stunting increasing from 31.3% in 1993 to 40.1% in 2008–09 (F(1, 1044) = 4.18, p < 0.041). However among girls aged 24–35 months, stunting declined significantly from 53.1% in 1993 to 43.1% in 2008–09 (F(1, 1017) = 9.88, p < 0.002). Analyses by province showed significant decreases in stunting prevalence for boys in Nyanza from 40.6% in 1993 to 30.8% in 2008–09 (F(1, 162) = 5.35, p < 0.022).

The trends by maternal education were not significant for most sub-groups except a decline in stunting among boys born to mothers with incomplete primary education, from 48.8% in 1993 to 41.5% in 2008–09 (F(1, 956) = 5.05, p < 0.025). By WI, most trends were not statistically significant, with the exception of a decline among boys living in households in the richer WI quintile (F(1, 717) = 5.98, p < 0.015).

While the overall national trend in stunting for boys and girls combined stagnated during the study period, girls’ prevalence seemed to have gotten worse in certain socio-demographic segments comparing 1993 versus 2008–09. Stunting prevalence was severe in 1993 and still increased by 2008–09 among girls aged 12–23 months (31.3% to 40.1%), girls born to mothers with no education (42.9% to 44.0%), girls born to mothers with complete primary education (31.8% to 34.7%), and girls belonging to the poorest (44.0% to 46.6%) and middle (34.4% to 39.6%), wealth quintiles.

### Trends in underweight

Table 4 provides the detailed trend analysis for underweight. The national trend for all children and separate trends for boys and girls showed significant declines in underweight. Underweight declined among boys and girls combined, from 19.7% in 1993 to 15.0% in 2008–9 (F(1, 1136) = 11.80, p < 0.001), among boys from 21.4% in 1993 to 16.4% in 2008–09 (F(1, 1136) = 7.96, p < 0.005), and among girls from 17.2% in 1993 to 12.8% in 2008–09 (F(1, 1136) = 7.24, p < 0.007). Age specific analysis showed significant declines among boys aged 0–5 months (F(1, 932) = 9.37, p < 0.002), girls aged 6–11 months (F(1, 925) = 4.09, p < 0.043), and boys aged 12–23 months (F(1, 1048) = 8.32, p < 0.004).

Provincial analyses showed significant declines in underweight among boys and girls in Nyanza. Boys’ prevalence reduced from 21.6% in 1993 to 14.0% in 2008–09 (F(1, 161) = 6.95, p < 0.009) and that for girls reduced from 20.9% in 1993 to 10.8% in 2008–09 (F(1, 161) = 10.39, p < 0.002). Boys and girls residing in rural areas recorded significant declines in underweight with boys’ levels reducing from 22.6% in 1993 to 17.1% in 2008–09 (F(1, 871) = 8.31, p < 0.004), and girls’ levels declining from 18.4% in 1993 to 13.8% in 2008–09 (F(1, 871) = 6.30, p < 0.012).

Most of the trend analyses of maternal education were not statistically significant. Only boys born to mothers with incomplete primary education showed a significant decline from 26.1% in 1993 to 19.4% in 2008–09 (F(1, 967) = 7.44, p < 0.006). There was a significant declining trend in underweight among boys in the poorest wealth quintile, from 31.7% in 1993 to 24.2% in 2008–09 (F(1, 551) = 5.40, p < 0.020) and among girls in the richer wealth quintile, from 15.2% in 1993 to 7.6% in 2008–09 (F(1, 716) = 4.26, p < 0.039). Comparison between the 1993 and 2008–09 surveys showed that prevalence of underweight dropped in 2008–09 in almost all sub-groups.

## Discussion

For each survey year, the wasting prevalence estimate was slightly lower for girls than for boys, which is consistent with previous studies from sub-Saharan Africa [34,35]. The overall national trend for wasting showed no significant change in the study period but there were important differences in the trends by age and sex. Older children aged 12–23 months showed a declining trend. Evidence on child growth patterns from many countries in the developing world shows that the prevalence of wasting is stable at all measurement points from about 12 months of age and on, after a six month period of sharply increasing wasting prevalence following weaning [36]. Therefore, the lessened risk of wasting over time observed in this study among Kenyan 12–23 month olds may be a result of improved post-weaning child care and feeding from the mid-1990’s on. This calls for closer investigation of archival data from KDHS and other sources on care and feeding patterns during the past two decades, to observe which care and feeding factors and trends may account for the reduction in wasting. The emphasis on overall care, and not just feeding, is in concert with recent conclusions that proper hygiene practices and access to adequate water, proper sanitation and reliable health services may be as important or even more important determinants of child growth than feeding practices [37].

As to sex differences, wasting among girls overall declined significantly, while remaining stable among boys. Yet some groups of boys did improve. Using a liberal criterion for significance of p < 0.10, the pattern of significant trends in wasting (12 trends as shown in Table 2) were all in the direction of improvement, observed predominantly in females. But trends in wasting also showed significant improvement among older boys and those living in Eastern and Nyanza provinces. The favourable trends in these provinces for both girls and boys are noteworthy, since Eastern province experiences marked perennial food shortages, while Nyanza is among the provinces with the highest poverty levels in Kenya [38,39]. Climate research in the Eastern province has observed no discernible increasing or decreasing trend either in the annual or seasonal rainfall from 1960’s to the present [40]. It seems unlikely that changing weather conditions might have resulted in improved local food production. In light of this, one possible explanation for the improved wasting trends is the impact of food security initiatives, such as the Kenya Special Programme [39]. However, returning to the theme that overall care may be as important as feeding care, evidence from many countries suggests the importance to child growth of policies in diverse arenas. These include immunization, safe water provision, female literacy, income distribution and support for agriculture [41]. Since it is unlikely that there is any single source within countries with expertise and information on all these features of social and political life, transdisciplinary research [42] seems essential to develop better appreciation of the factors that underpin the trends in child growth reported here.

Similar to wasting, trends in stunting at a national level remained stagnant. However, stratification by sex showed a decline among boys. The high prevalence in stunting among boys as compared to girls is in agreement with the literature on stunting in sub-Saharan Africa [11], but the improvement over time in boys, more so than in girls, is difficult to explain. Looking to family dynamics, the literature on parental sex bias in relation to child care and feeding practices is contradictory and the evidence for bias is scarce [12,13,35]. DHS data have been brought to bear on this subject, but only via indirect inferences based on parental education differences [35]. Due to data limitations, the DHS, and most other survey data for that matter, may be inadequate for direct investigations of social and psychological factors underlying sex differences in child growth. Supporting this view is Marcoux’s meta study of 306 child nutrition surveys from across the developing world, of which 74 percent showed no sex differences in wasting, stunting and underweight [43]. That sex differences are difficult to detect reliably in survey research recommends against the use of the survey study design in the search for factors underlying sex differences in child growth. Mixed methods studies of cohorts, and of cases and controls, may be more illuminating.

Analyses by age showed stunting to be relatively lower in younger children and increased with age, in line with other research evidence that the prevalence of stunting increases with age [44]. The comparatively low and stable prevalence posted by children in the youngest age category (0–5 months) is likely due to stable childcare and feeding practices during the pre-weaning stage of development. Actually, in Kenya exclusive breastfeeding increased from 12.7% in 2003 to 31.9% in 2009, while early complimentary feeding at the age of 2–3 months decreased from 81% in 1993 to 32% by 2008 [7]. That stunting in this age group did not show a decline is likely due to a ‘floor effect’, with near lowest feasible levels of stunting already achieved by the mid-1990’s.

The high levels of stunting among children above 12 months and the increasing trend in stunting among girls aged 12–23 months indicates the seriousness of stunting, which seems to manifest itself at the onset of complimentary feeding. Studies have shown that foods used to compliment breastfeeding in Kenya are of low nutritive value [45]. The most preferred porridge is made of composite flours causing negative nutrient-nutrient interactions and also causing mal-absorption due to the child’s immature gut. Such foods are also high in anti-nutrients such as phytates and tannins that bind available nutrients and thus reduce bioavailability [45]. Further research is needed to explore the possibility that the nutritive value of the food served to girls in this age segment has worsened over the study period. The significant improvement among older children, especially among girls aged 24–35 months, could be an indication of older girls responding better to nutritional interventions leading to catch up growth [36], but more research is needed to investigate this issue.

The significant improvements in stunting levels in Nairobi could be attributed to the accrued social-economic and infrastructural advantage enjoyed in the capital region in terms of the number of health facilities and personnel, higher literacy levels and better economic performance [46]. As in the case of wasting, the improvement in stunting among boys in Nyanza province was unexpected due to its high incidence of poverty. Nevertheless, Nyanza province has witnessed an increase in literacy levels [38] and this could be one of the contributing factors to better growth, as maternal literacy is associated with reduced risk of stunting [47,48].

Higher socio-economic status is associated with better utilization of health care services, better access to food of high quality and quantity, better nutrition, improved sanitation and household possessions [49,50]. This advantage was observed in the present study, with a significant reduction in stunting observed among children in rich households but not in poor ones. The public health significance of this pattern is alarming, even though Kenya experienced a decrease in the percentage of people living in poverty. It is estimated that the number of people living below the poverty line increased from 13.4 million in 1997 to about 16.6 million in 2006 [51], increasing the number of children in poor households and at risk of stunting.

Overall, the results show that faltering child growth remains a significant public health challenge in Kenya. It is beyond the present scope to undertake an analysis of causes of faltering growth in the Kenyan context. Here, we must be content merely to point to the complexity of the causal landscape, and the need for research that goes beyond the simple descriptive analyses presented in this paper. Among the critical causes of faltering child growth are poor agricultural performance and food distribution at a macro level, and micronutrient deficiency at the level of individuals. At the macro level, there was a worrying decline in productivity in the Kenyan agricultural sector from a real growth rate of 4.4% in 1996 to −5.4% in 2008. This poor performance translates to less food for the fast-growing Kenyan population, poor economic returns as a result of a decline in agricultural export earnings, and increased unemployment due to the decrease in household farm incomes [46,50,52].

At the individual level, dietary zinc in particular is essential in bolstering immunity, protein metabolism and linear growth, and its deficiency precipitates retarded growth [49]. Bwibo and Nuemann observed that food served to Kenyan children has multiple micronutrient deficiencies, placing child at risk of poor growth regardless of the quantity of food provided by the agricultural sector [45].

This span from macro to micro level causal factors illustrates the complexity of the causal web that underlies faltering child growth, to which an array of sub-optimal childcare practices and inadequate access to health care also contribute. This complexity is signaled strongly in the present findings that compare child growth trends in urban and in rural areas. While the terms ‘urban’ and ‘rural’ are demographic concepts referring to population number and density, urban versus rural living conditions include important variation in social factors, such as rates of unemployment and illiteracy, access to health facilities, and household and community poverty level [53]. The declining trends in underweight in rural areas as compared to stagnant trends in urban areas underscore the possibility that urban areas are experiencing a decline in their perceived advantage over rural areas [54]. The high urbanization rate brought about by rural–urban migration has significantly reduced the infrastructural advantage urban areas used to enjoy, and has resulted in increased urban poverty. While hardcore poverty declined in rural areas from 34.8% in 1997 to 21.9% in 2005/06, it increased marginally in urban areas from 7.6% in 1997 to 8.3% in 2005/06 [46]. Further research on urban/rural child growth patterns should therefore be complemented by studies of changing urban and rural living conditions, so that the context of child growth is better appreciated. This should include differentiation between urban areas generally and those in capital regions such as Nairobi, which may enjoy special advantages due to proximity to central government.

### Study limitations

The more data points over time the more robust the trend analysis. With just the four data points (1993, 1998, 2003 and 2008–09) available for the present analyses, we treat our findings and interpretations with due caution. However, we are not aware of any other data on child undernutrition in Kenya with more than four data points over time, and therefore consider the present effort defensible in the interest of providing the best trend estimates possible with the limited data now available.

While decomposed analysis enables detailed understanding of trends within socio-demographic groups, it results in reduction in sample size. Trends in certain groups may fail to reach statistical significance, not necessarily due to lack of changes in prevalence, but rather to limited sample size giving rise to wider confidence intervals around prevalence estimates. It is not only relatively small sample size, but also sample size variation, that may hinder comparison of trends across socio-demographic groups. For example, two sub-group trend analyses with identical prevalence estimates at four points in time may be judged statistically significant in the sub-group with a relatively large n and insignificant in the sub-group with a relatively smaller n. In surveys wherein the sampling design has not included sampling strata at the level of socio-demographic sub-groups, such as the DHS, the limitations associated with variable n that were encountered in this study cannot be overcome.

An alternative to the decomposition approach (stratification) we have taken is to use multivariate analysis to control statistically for population composition changes over time, and to control for confounding and for effect modification (interactions amongst risk and protective factors). While statistically elegant, the main value of such multivariate analyses is to produce equations that predict future changes in outcome variables as a function of hypothesized changes in risk and protective factors. This approach has less utility for policy work than the decomposition approach, which produces more easily digestible information about sub-group trends. Nevertheless, it is a limitation of the present study that the multivariate relationships among the factors defining socio-demographic sub-groups have not been taken into account in the analyses of undernutrition prevalence.

## Conclusions

The national trends in childhood undernutrition in Kenya showed significant declines in underweight, but trends in wasting and stunting were stagnant. Analyses disaggregated by demographic and socio-economic segments revealed some departures from the overall trends. There were more declines in wasting among girls than boys in the various socio-demographic stratifications studied, and the opposite was true for stunting, with boys posting more declining trends compared to girls. These findings support the importance of conducting trend analyses at disaggregated levels within countries, if findings are to be useful in informing public health policy and the development of better-targeted childcare interventions. Concerted efforts should be made by relevant stakeholders to reduce the stagnating trends of undernutrition, especially for stunting, which has consistently remained high in most socio-demographic segments in Kenya.

## Endnotes

^a^Since the promulgation of the new constitution in 2010, provinces were renamed as regions.

^b^The KDHS is one of the MEASURE DHS projects in developing countries that collect data on important health indicators. It is a collaboration involving the Kenya National Bureau of Statistics, National AIDS Control Council, Ministry of Public Health and Sanitation, Kenya Medical Research Institute, National Coordinating Agency for Population and Development, ICF Macro, The United States Agency for International Development (USAID) and other non-governmental organizations.

## Abbreviations

WI: Wealth index; KDHS: Kenya demographic and health survey; SPSS: Statistical package for the social sciences; SD: Standard deviations; WHZ: Weight-for-height Z-score; WLZ: Weight-for-length Z-score; HAZ: Height-for-age Z-score; LAZ: Length-for-age Z-score; WAZ: Weight-for-age Z-score; DHS: Demographic and health survey.

## Competing interests

The authors declare that they have no competing interests.

## Authors’ contributions

DJM and MBM conceived the study, DJM conducted data analysis, interpretation of results and drafting of the manuscript, and MBM and KDMD revised the manuscript. All authors read and approved the final manuscript.

## Pre-publication history

The pre-publication history for this paper can be accessed here:

http://www.biomedcentral.com/1471-2431/14/5/prepub
